# The Physicochemical Properties of Starch Are Affected by *Wx^lv^* in *Indica* Rice

**DOI:** 10.3390/foods10123089

**Published:** 2021-12-13

**Authors:** Linhao Feng, Chenya Lu, Yong Yang, Yan Lu, Qianfeng Li, Lichun Huang, Xiaolei Fan, Qiaoquan Liu, Changquan Zhang

**Affiliations:** 1 Jiangsu Key Laboratory of Crop Genomics and Molecular Breeding/State Key Laboratory of Hybrid Rice/Jiangsu Key Laboratory of Crop Genetics and Physiology, College of Agriculture, Yangzhou University, Yangzhou 225009, China; linhaofeng123@126.com (L.F.); qqliu@yzu.edu.cn (Q.L.); 2 Key Laboratory of Plant Functional Genomics of the Ministry of Education, Jiangsu Co-Innovation Center for Modern Production Technology of Grain Crops, College of Agriculture, Yangzhou University, Yangzhou 225009, China; luchenyalcy@gmail.com (C.L.); yongyang951208@gmail.com (Y.Y.); luyan1210@163.com (Y.L.); qfli@yzu.edu.cn (Q.L.); lchuang@yzu.edu.cn (L.H.); fxl19820201@126.com (X.F.)

**Keywords:** rice, *Wx*, grain quality, amylose, starch structure

## Abstract

Amylose largely determines rice grain quality profiles. The process of rice amylose biosynthesis is mainly driven by the waxy (*Wx*) gene, which also affects the diversity of amylose content. The present study assessed the grain quality profiles, starch fine structure, and crystallinity characteristics of the near-isogenic lines Q11(*Wx^lv^*), NIL(*Wx^a^*), and NIL(*Wx^b^*) in the *indica* rice Q11 background containing different *Wx* alleles. Q11(*Wx^lv^*) rice contained a relatively higher amylose level but very soft gel consistency and low starch viscosity, compared with rice lines carrying *Wx^a^* and *Wx^b^*. In addition, starch fine structure analysis revealed a remarkable decrease in the relative area ratio of the short amylopectin fraction but an increased amylose fraction in Q11(*Wx^lv^*) rice. Chain length distribution analysis showed that Q11(*Wx^lv^*) rice contained less amylopectin short chains but more intermediate chains, which decreased the crystallinity and lamellar peak intensity, compared with those of NIL(*Wx^a^*) and NIL(*Wx^b^*) rice. Additionally, the starches in developing grains showed different accumulation profiles among the three rice lines. Moreover, significant differences in starch gelatinization and retrogradation characteristics were observed between near-isogenic lines, which were caused by variation in starch fine structure. These findings revealed the effects of *Wx^lv^* on rice grain quality and the fine structure of starch in *indica* rice.

## 1. Introduction

The main edible part of rice is the endosperm, which is mainly composed of starch, accounting for more than 80% of milled rice. Starch in the rice endosperm is generally divided into two types, amylose and amylopectin. Rice quality performance, such as eating and cooking quality, and nutrition quality, is mainly determined by the rice grain composition and the fine structure of starch [1,2]. Generally, rice grains with medium/low amylose contents have a soft texture (better taste), while rice grains with high amylose exhibit a firm texture (bad taste) [3]. However, from a global respective, the assessment of rice grain quality is a challenge, because rice that is desirable to one group of people may not be to another [1]. For example, consumers in southern China, India, and Pakistan prefer long slender grains with a relatively harder texture, whereas consumers in northern China, Japan, and South Korea prefer medium grains with a relatively soft texture [4]. In addition, from the perspective of nutritional quality, in cooked rice, amylose molecules generally reassociate rapidly and thus form a precipitate or gel upon cooling, which resists digestion [5]. Therefore, rice grains with a high amylose level tend to be healthy for humans. 

The synthesis of rice starch involves a series of enzymes that are involved directly in starch synthesis and transcription factors that regulate the process indirectly. Among them, the *Wx* gene encodes granule-binding starch synthase (GBSSI, the main enzyme involved in amylose synthesis in the rice endosperm. Studies have found that functional single nucleotide polymorphisms (SNPs) in *Wx* are associated with variations in the amylose content (AC) in rice cultivars. To date, a series of *Wx* alleles have been identified, including *Wx^lv^*, *Wx^a^*, *Wx^in^*, *Wx^b^*, *Wx^mw/la^*, *Wx^mp^*, *Wx^mq^*, *Wx^op/hp^*, and *Wx* [6,7,8,9,10,11,12]. In normal rice cultivars, a high AC level (> 25%) is determined by the *Wx^a^* and *Wx^lv^* alleles; however, the *Wx^lv^* allele was identified as the ancestral type of the *Wx* gene, contributing to a higher AC than that of the *Wx^a^* allele. Moreover, this allele also contributes to a very low starch viscosity and a soft gel consistency because of ungelatinized starch granules [6]. As regards the other *Wx* alleles, *Wx^in^* results in an intermediate AC (about 20%); *Wx^b^* contributes to a low AC (about 15%); *Wx^mw/la^* also contributes to a low AC (about 14%); *Wx^mp^*, *Wx^mq^*, and *Wx^op/hp^* contribute to a very low AC (8–12%) [6,11,12,13]. For the *waxy* rice, generally, the 23-bp deletion in the *Wx* locus (*wx* allele) is responsible for glutinous rice [14]. Accumulated studies have shown that all the alleles had been selected in response to cultural preferences around the world [1,6]. Among them, *Wx^lv^* is distributed mainly in wild rice and aus subspecies, whereas *Wx^a^* is distributed mainly in *indica* rice subspecies, and *Wx^b^* is distributed mainly in japonica rice subspecies [6]. 

There has been great progress made in identifying natural allelic variations in the *Wx* gene and studying their expression profiles (enzyme activities) and relationships with AC using natural rice varieties, near-isogenic lines (NILs), and chromosome segment substitution lines [9,15,16,17]. Moreover, studies showed that *Wx* allelic variation contributes to AC variation, but also causes changes in the amylopectin fine structure [18,19]. However, there is no accurate information about the effect of high AC-type *Wx* alleles (e.g., *Wx^lv^*) on the fine structure and physicochemical properties of rice starch. 

In our previous work, we constructed several NILs for the *Wx* locus in both japonica and *indica* rice backgrounds, and we investigated the effects of *Wx^lv^* on rice grain cooking and eating quality profiles in the japonica rice background; however, limited information is available in the *indica* rice background, because we only constructed one NIL(*Wx^a^*) in the background of *indica* rice Q11(*Wx^lv^*) [6]. To obtain more information about the effects of *Wx* allelic variation on rice grain quality profiles, we further constructed a NIL of the *Wx^b^* allele, NIL(*Wx^b^*), in the same *indica* background. Thus, this study aimed to compare the allelic variation of these *Wx* alleles, especially *Wx^lv^*, taking into account rice grain physicochemical properties in the *indica* rice background. In addition, we investigated the starch fine structure profiles during the development of the endosperm. The results of the present study will deepen our knowledge concerning the influence of *Wx* allelic variation on the physicochemical properties and starch structure of *indica* rice grains.

## 2. Materials and Methods

### 2.1. Plants and the Preparation of Samples

The NIL(*Wx^a^*) on a background of *indica* cultivar Q11 (carrying the *Wx^lv^* allele) was generated by introgressing the *Wx^a^* allele from *indica* cultivar Guichao 2 after six backcrosses [6]. Similarly, the *Wx^b^* allele, harbored by *indica* cultivar 9311, was introgressed after six backcrosses using marker-assisted selection to generate NIL(*Wx^b^*). The three rice lines Q11(*Wx^lv^*), NIL(*Wx^a^*), and NIL(*Wx^b^*) were cultivated at an experimental farm of the Yangzhou University (Jiangsu, China). The three lines were each grown randomly in triplicate, during summer, and under the same management and climatic conditions.

For rice grain quality measurement, white rice was obtained from mature seeds by harvesting, dehusking, milling, and screening, as described previously [12]. A portion of the milled grains was subjected to milling using a FOSS 1093 Cyclotec Sample Mill (Foss Tecator, Hoganas, Sweden) with a 0.5 mm sieve. Another portion of the milled grains was subjected to starch isolation via the previously described alkaline protease method [12]. To measure the starch structure of rice grains during endosperm development, immature seeds were freshly harvested at 5, 10, 15, 20, and 25 days after flowering (DAF). Endosperms were freeze dried and ground into a powder in liquid N_2_. The immature endosperm powders were then subjected to starch isolation using the alkaline protease method.

### 2.2. Analysis of Rice Grain Composition

The moisture content (MC) was determined using a halogen moisture analyzer (Mettler Toledo MJ33, Greifensee, Switzerland). Briefly, 1 g of sample was weighed in the weighing pan, incubated at 120 °C for 10 min, and then the water loss rate was calculated via auto calculation by the instrument. A Kjeltec 2300 nitrogen determination instrument (Foss Tecator) was used to determine the rice flour crude protein content (PC) from different NILs. The apparent amylose content (AAC) was measured colorimetrically as described by Zhang et al. [12]. The gel consistency (GC) was measured as described by Tan et al. [20]. A total starch assay kit (K-TSTA; Megazyme, Wicklow, Ireland) was used to measure the rice flour total starch content (TSC) according to the method of Zhu et al. [21].

### 2.3. Analysis of Rice Pasting and Thermal Profiles

A Rapid Visco Analyzer (RVA) (Techmaster, Newport Scientific, Warriewood, Australia) was used to assess the pasting properties of rice starch and flour, as described previously [18]. The RVA parameters were extracted from the viscosity curve and included the peak time (PT), setback viscosity (SBV), breakdown viscosity (BDV), cool paste viscosity (CPV), hot paste viscosity (HPV), and peak viscosity (PKV). 

Differential scanning calorimetry (DSC, DSC200F3, Netzsch Instruments NA LLC, Burlington, MA, USA) was used to determine the thermal properties as described previously [21]. Briefly, native rice starches (5 mg) were added to a sealed aluminum pan, and then 10 μL of deionized water was added. The samples were allowed to equilibrate for 1 h at room temperature before measurement. Then, samples were heated from 15 to 120 °C at 10 °C/min. To measure retrogradation, the gelatinized starch-containing DSC pans were stored for 1 week at 4 °C, followed by rescanning under the same conditions. The DSC parameters calculated from the DSC curve were the enthalpy of gelatinization (Δ*H*), conclusion temperature (*T*_c_), peak temperature (*T*_p_), and onset temperature (*T*_o_). Triplicate measurements were performed for all analyses. 

### 2.4. Analysis of the Fine Structure of Starch

Isoamylase (EC3.2.1.68, E-ISAMY, Megazyme, County Wicklow, Ireland) was used to debranch rice starch, as described previously [12]. Gel permeation chromatography (GPC) was used to determine the distribution of debranched starches’ relative molecular weight using an Agilent PL-GPC 220 system (Polymer Laboratories Varian, Inc., Amherst, MA, USA) as described previously [21]. Molecular weight distribution graphs were drawn using GPC data from standard dextrans of know molecular weights (Mw: 3650; 20,100; 131,400; 610,500; 1,185,000; 3,450,000; 5,900,000, and 6,300,000) which were purchased from the American Polymer Standards Corporation (Mentor, OH, USA). For GPC measurement, 3 mg of debranched starch was dissolved in 2 mL dimethyl sulfoxide (DMSO) solution with 5 mM NaNO_3_, and heated in a metal bath at 85 °C for 12 h. Thereafter, the sample was left to cool to room temperature, centrifuged at 4000× *g* for 10 min, and then 1.5 mL of the supernatant was transferred to a sample vial. For each sample, 100 μL of the solution was injected into the GPC system by the autosampler. The mobile phase used for the GPC system was also DMSO, and the flow rate of the measurement was 0.8 mL/min. The GPC data were subjected to detection, recognition, and identification (DRI) analyses, and the molecular size distributions were reported as the dextran-equivalent molecular weight (Mw). The molecular weight distribution curves were analyzed, and the following GPC parameters were extracted: amylopectin (AP), amylopectin short chains (AP1), amylopectin long chains (AP2), and amylose chains (AM). Each of the fractions was calculated using the total integrated area of each GPC peak. Besides, quantitative analysis of debranched starch was also carried out following the method of Zhu et al. [21] in a high-performance anion-exchange chromatography (HPAEC) system (Thermo ICS-5000, Thermo Corp, Sunnyvale, CA, USA) incorporating a pulsed amperometric detector, a guard column, a CarboPacTM PA200 analytical column, and an AS-DV autosampler. 

### 2.5. Analysis of the Crystalline Structure of Starch

X-ray diffraction (XRD) (D8 ADVANCE X-ray diffractometer, Bruker, Karlsruhe, Germany) was used to analyze the starch supra-molecular structure according to a previously described method [18]. From the XRD curve, the relative crystallinity (RC) was determined using the following equation:

RC = Ic/(Ia + Ic)

where Ia represents the non-crystalline area proportion, and Ic represents the crystalline area proportion in the diffraction profile. The starch crystal lamella structure was determined with the aid of a Bruker Nano Star small-angle X-ray scattering (SAXS) instrument with a Vantec 2000 detector and pinhole collimation for point focus geometry, as described previously [22]. The position of the peak (*q*o) was used to calculate the SAXS parameter, Bragg spacing (D), which represents the lamellar distance, using D = 2π/*q*o. 

### 2.6. Statistical Analysis

Three technical replicates were performed for all measurements in this study, and all data are shown as means ± the standard deviation (SD). SPSS 16.0 statistical software (IBM Corp., Armonk, NY, USA) was used to carry out a one-way analysis of variance (ANOVA) on the data. Statistical significance was accepted at *p* < 0.05.

## 3. Results and Discussion

### 3.1. Rice Grain Quality Profiles 

To investigate the effect of *Wx* alleles on rice grain phenotype, we assessed the appearance of brown and milled grains from Q11(*Wx^lv^*), NIL(*Wx^a^*), and NIL(*Wx^b^*). All three rice lines showed a very similar grain size and appearance (Figure S1). The physicochemical characteristics and major components of rice flours were then assessed. Rice flours showed no significant differences in the MC, PC, and TSC values (Table 1). However, rice flour from Q11(*Wx^lv^)* showed the highest AAC among the three samples, which was consistent with our previous results that rice carrying *Wx^lv^* tends to have a higher AAC than those carrying other *Wx* alleles [6]. It is generally accepted that the GC correlates negatively with AAC, and rice with a high AAC will have a hard GC [18,23]. However, rice flour from Q11(*Wx^lv^*) had the highest AAC level and exhibited a very soft GC, compared with the other lines. In fact, previous studies proposed that rice varieties with this characteristic of high AAC but soft GC were good germplasm resources for rice grain quality improvement [24,25]. However, this might not be true, because we have shown that starch from rice carrying *Wx^lv^* cannot be gelatinized completely during heating, and thus the gelatinized paste contains many partially swollen granules, resulting in high flowability (higher GC) [6]. Moreover, rice grains with this high AAC but soft GC phenotype exhibited poor eating quality [6].

### 3.2. Starch Fine Structure

**Figure 1:**
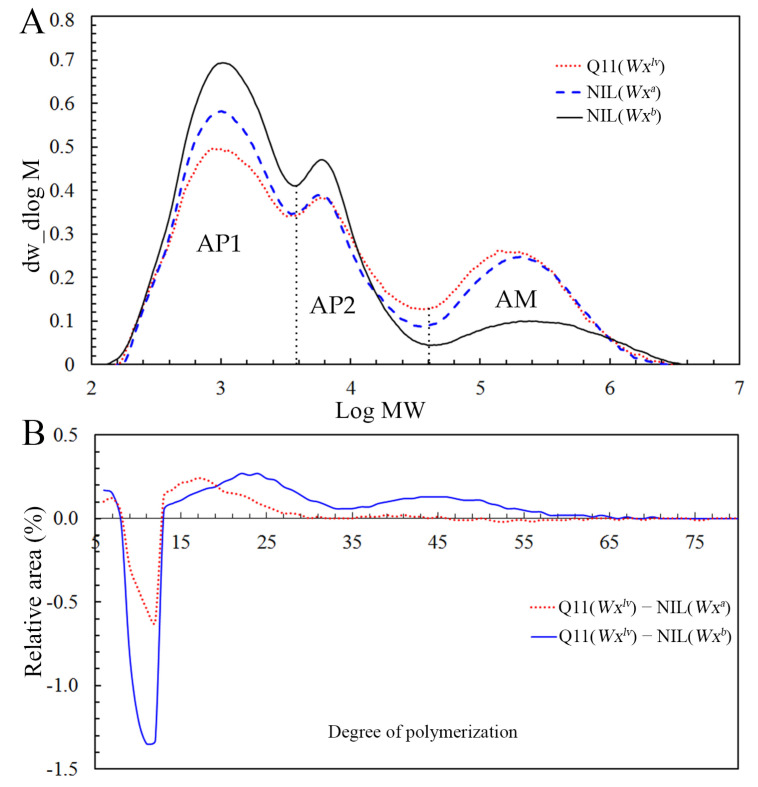
GPC and HPAEC determination of starch fine structure: (**A**) isoamylase-debranched starch relative molecular weight distributions; (**B**) changes in the chain length distribution of amylopectin. GPC, gel permeation chromatography; HPAEC, high-performance anion-exchange chromatography; Mw, weight average molecular weight; AP1, amylopectin with short branched chains; AP2, amylopectin with long branched chains; AM, amylose.

GPC was used to determine debranched starches’ relative molecular weight distribution. As shown in Figure 1A, three peaks (high, medium, and low) were observed, which were named AP1, AP2, and AM, respectively. Generally, the true amylose content is represented by the ratio of the AM area to the total area, and the ratio of AP1 to AP2 represents the extent of amylopectin branching. Compared with the starch from NIL(*Wx^a^*) and NIL(*Wx^b^*) rice, Q11(*Wx^lv^*) rice starch showed a significantly lower AP1 peak, whereas the AM peak was the highest among the three samples. We further compared the ratio of each fraction, in which starch from Q11(*Wx^lv^*) rice exhibited the lowest AP1 fraction among the three samples (Table 2). Moreover, the AP2 fraction of Q11(*Wx^lv^*) starch was higher than the AP2 fraction of NIL(*Wx^a^*) starch. In terms of the branching degree, Q11(*Wx^lv^*) rice starch showed the lowest degree of branching, which was consistent with our previous results that rice with higher AC tended to have a low branching degree [18]. Moreover, in agreement with the AAC data, starch from Q11(*Wx^lv^*) rice showed a relatively higher AM fraction (Figure 1A, Table 2). *Wx^lv^* NIL-derived starch in the japonica rice background has an increased AM fraction, especially short-chain AM [6]. These findings demonstrated that *Wx^lv^* is involved in amylose chain biosynthesis and also has a role in amylopectin biosynthesis. 

The chain length distribution (CLD) of the branched starch from the three rice lines was further analyzed using HPAEC. Generally, amylopectin chains can be classified as A chains (DP 6–12), B1 chains (DP 13–24), B2 chains (DP 25–36), and B3+ chains (DP ≥ 37) according to the classical amylopectin cluster model [26]. The rice starch from Q11(*Wx^lv^*) comprised significantly lower levels of A chains (DP 6–12) but higher levels of B chains (DP ≥ 12), compared with those in NIL(*Wx^a^*) (Figure 1B). Similarly, the Q11(*Wx^lv^*) starch exhibited lower numbers of A chains, and higher numbers of B chains, to that of NIL(*Wx^b^*) rice; however, the degree of change was relatively high. Similarly, previous studies in maize and rice found that the *Wx* gene led to changes in both amylose and amylopectin fine structures [18,19,27]. The above results suggested that *Wx* functions to determine the amylopectin molecular structure, at least in part.

### 3.3. Starch Fine Structure in the Developing Endosperm

**Figure 2:**
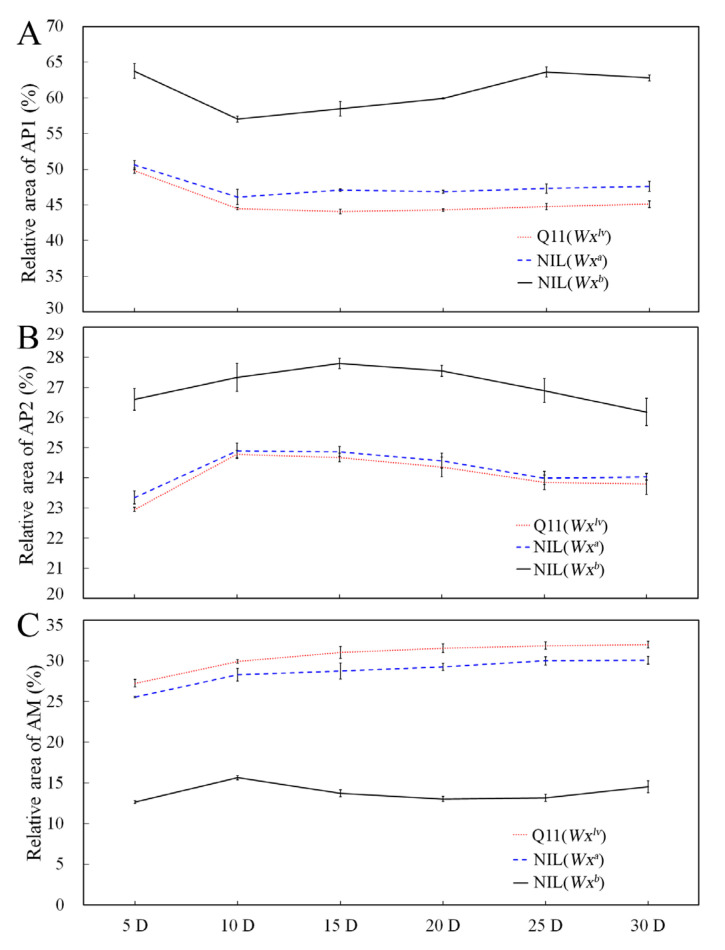
Changes in starch fraction distribution of near-isogenic line (NIL)-derived rice grains at various developmental stages. Panel (**A**) shows the AP1 fraction distribution of starch during the filling stage. Panel (**B**) shows the AP2 fraction distribution of starch during the filling stage. Panel (**C**) shows the AM fraction distribution of starch during the filling stage. AP1, amylopectin with short branched chains; AP2, amylopectin with long branched chains; AM, amylose; D, days after flowering.

To further clarify the changes in the starch fine structure in the developing endosperm, starch was extracted from grains at various DAF. The isoamylase-debranched starch was used for GPC measurement, and fractions of AM, AP1, and AP2 were calculated and compared. The GPC profiles are shown in Figure 2. All the starches showed a relatively higher amount of the AP1 fraction at 5 DAF, which decreased quickly at 10 DAF (Figure 2A). However, from 10 DAF, all the samples showed an increased AP1 fraction during the development of the endosperm. Similar to the results for mature rice grains, starch from NIL(*Wx^b^*) rice had a significantly higher AP1 fraction throughout endosperm development. In addition, Q11(*Wx^lv^*) and NIL(*Wx^a^*) starch showed similar AP1 levels at the early stage of endosperm development (5 and 10 DAF); however, from 15 DAF, Q11(*Wx^lv^*) starch exhibited the lowest AP1 fraction, compared with the other samples. In terms of the AP2 fraction, in the three rice lines, little change was observed during endosperm development (Figure 2B), which was consistent with a previous study [28]. Starch from NIL(*Wx^b^*) rice also showed a relatively higher AP2 fraction throughout endosperm development, whereas there were no significant changes in the AP2 fraction between Q11(*Wx^lv^*) and NIL(*Wx^a^*) before 15 DAF. From 20 DAF, the AP2 fraction in Q11(*Wx^lv^*) starch increased to a greater extent than that of NIL(*Wx^a^*) starch. Comparison of the AM fractions showed that Q11(*Wx^lv^*) starch contained relatively high amounts of AM throughout the whole filling stage (Figure 2C). 

Starch accumulation is crucial for the development of the rice endosperm. It is well known that the accumulation of starch increases rapidly with the grain filling process, and this process is sensitive to the environmental temperature [28,29]. However, only a few studies have focused on the accumulation of various components of starch during rice grain filling [30]. ln fact, studies have found that the synthesis of amylose and amylopectin does not occur completely at the same time, because amylose deposition occurs in the pre-existing starch granule matrix, and a water-insoluble amylopectin scaffold is required to target GBSS to the granule [31]. Thus, it makes sense that the accumulation of AP1 abundantly occurs in the early stage of grain development. Furthermore, although the three starch fractions were significantly different between NILs at the same filling stage, the accumulation trends of these fractions were the same during the filling process. It should be noted that the accumulation of the AM fraction from Q11(*Wx^lv^*) starch was the highest among the three rice lines during the whole filling stage, which could be attributed to its higher GBSSI enzyme activity, as reported previously [6]. Moreover, our data showed that the differences in amylose content appeared in the early stage of grain development and continue to the mature stage, which agreed well with the gradually increased expression profiles of the *Wx* gene during the filling stage [6]. 

### 3.4. Crystalline Structure of Starches from Different NILs 

Starch’s semi-crystalline layer comprises crystalline and amorphous regions arranged as lamellae, with a repeating distance of 90–100 Å. Moreover, amylopectin branched chains form double helices, thus determining the crystalline region, in which the amylopectin branched chains are arranged horizontally into a lattice [32]. It is believed that the short-range ordered helices form double helices, while the long-range ordered helices form a crystalline structure. Herein, XRD was employed for long-range ordered analysis of starch. As shown in Figure S2A, the XRD patterns of the starches from the three rice lines were very similar, showing major peaks of diffraction at approximately 15° and 23° 2θ. Thus, as in the majority of normal cereal starches, all samples displayed a typical type A diffraction pattern [33]. However, the XRD parameters were different among the three samples. Specifically, starch from Q11(*Wx^lv^*) rice had the lowest RC, while NIL(*Wx^b^*) starch showed the highest RC among the three samples (Table 3). It is believed that the degree of starch crystallinity correlates positively with the ratio of long amylopectin. In addition, amylose destroys the crystal accumulation of amylopectin. Thus, compared with the other NILs, Q11(*Wx^lv^*) had a higher amylose content and a lower number of longer chains, resulting in lower relative crystallinity.

SAXS was used to assess the starches’ supramolecular structure, with the aim of determining whether starch structural changes affect the ordered structure of starch. Similar single peaks were observed in all three samples (Figure S2B); however, the peak intensities differed. The peak position was derived from the alternate arrangement of amylopectin crystalline and non-crystalline lamellas, which correspond to the repeating distance of the lamellar. Moreover, the difference in peak intensity is caused by electron density differences between the amorphous region and the lamellae. The SAXS curves were used to determine the average lamellar distance (D) and lamellar peak intensity (Imax) (Table 3). Q11(*Wx^lv^*) starches had significantly lower Imax values, while the D value was higher in Q11(*Wx^lv^*) among the three samples. It is believed that short chains of amylopectin form ordered semi-crystalline structures, the number of which determines the Imax value [32]. Our previous study showed that the AP1 fraction correlated positively with the Imax value [34]. Thus, variation in the Imax value among the three samples might reflect differences in their AP1 fractions. In terms of the differences in D values, the amylose content might be a key reason for the higher D of Q11(*Wx^lv^*) starch, because the lamellar distance has been proven to correlate positively with the amylose content.

### 3.5. Rice Flour and Starch Pasting Properties

**Figure 3:**
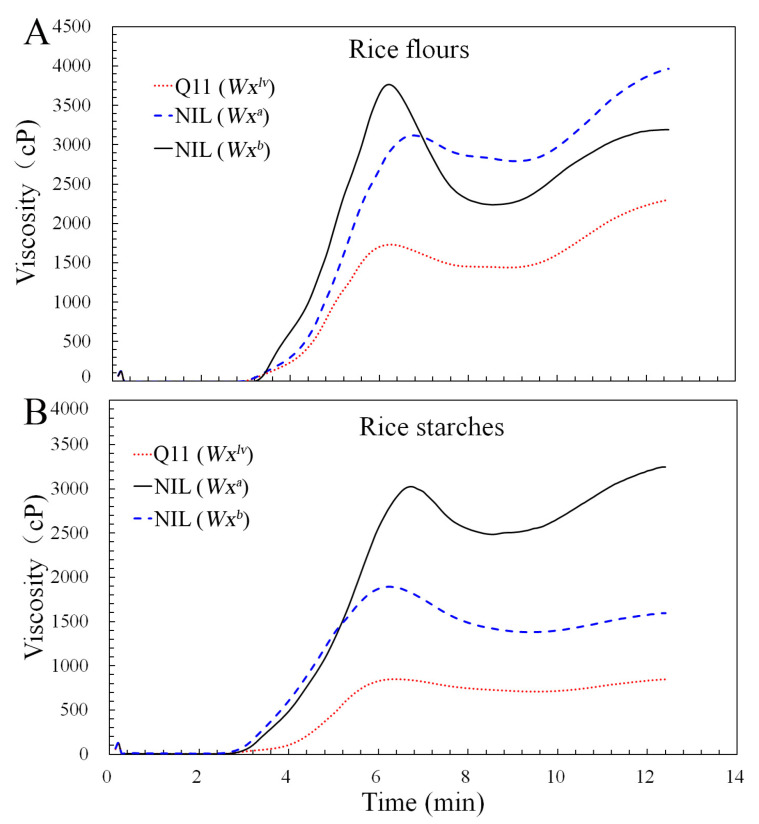
Rapid Visco Analyzer (RVA) patterns of rice flours and starches from different NILs. Panel (**A**) shows RVA patterns of rice flours. Panel (**B**) shows RVA patterns of rice starches.

The *Wx^lv^* was cloned initially based on the very low starch viscosity curve measured by RVA in the japonica rice background [6]. To further investigate the effects of this allele on rice pasting profiles in the *indica* rice background, rice flour and starch from the three lines were used for RVA analysis. As shown in Figure 3A, rice flour from Q11(*Wx^lv^*) exhibited a markedly lower RVA curve than those of NIL(*Wx^a^*) and NIL(*Wx^b^*), which was consistent with the results in the japonica background [6]. Moreover, the RVA parameters were calculated from the RVA curves (Table S1). The PKV (839.00), HPV (708.50), BDV (130.50), and CPV (835.5) of Q11(*Wx^lv^*) rice flour were significantly lower than those of the NIL(*Wx^a^*) (1892.00, 1377.00, 515.00, and 1592.50, respectively) and NIL(*Wx^b^*) (3022.50, 2482.50, 540.00, and 3244.50, respectively) flours. Moreover, the Q11(*Wx^lv^*) rice flour showed lower SBV (−3.50) and PT (6.33) values than the NIL(*Wx^b^*) flour (222.00, and 6.73, respectively) but higher values than NIL(*Wx^a^*) flour (−299.50, and 6.20, respectively). To determine if the RVA curve was affected by the starch structure, the RVAs of starch samples were determined. Similarly, lower RVA curves and parameters including PKV, HPV, BDV, and CPV were observed for Q11(*Wx^lv^*) starch, compared with those of the other samples (Figure 3B, Table S1). In addition, we observed that the RVA profiles of NIL(*Wx^a^*) and NIL(*Wx^b^*) were different between rice flour and starch, which indicated that factors within rice flour affect the RVA parameters [35]. Previous studies have shown that when the AC reaches more than 40%, the end temperature of starch gelatinization will be greater than 100 °C, leading to incomplete gelatinization using the standard RVA test; therefore, it shows a much lower viscosity [36]. However, unlike high AC rice, Q11(*Wx^lv^*) rice showed a normal high AC level. It has been reported that a higher PKV can result from a lower AC [27,37]. Generally, the amorphous regions of amylose molecules can form double helices, leading to starch granule stabilization to prevent thermal expansion, thereby reducing its ability to inhibit starch swelling and leading to greater swelling and higher PKV. Thus, the higher AC in Q11(*Wx^lv^*) rice is the main reason for the low PKV.

### 3.6. Rice Starch Thermal Properties

**Figure 4:**
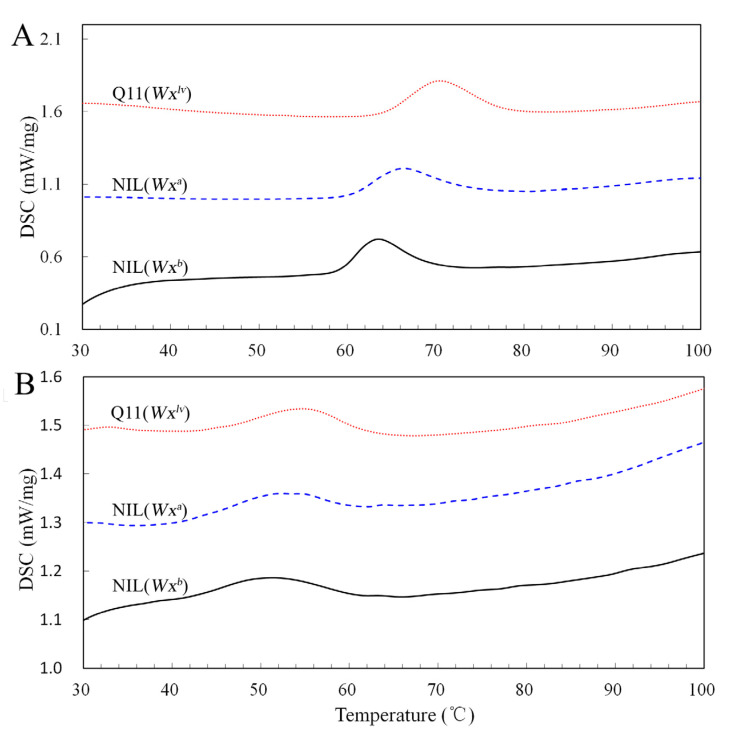
Differential scanning calorimetry (DSC) of NIL-derived rice starches. Panel (**A**) shows the gelatinization curves of native starches. Panel (**B**) shows the retrogradation curves of gelatinized starches.

DSC was employed to determine the NIL-derived starch gelatinization characteristics associated with *Wx* allelic variation. Q11(*Wx^lv^*) and NIL(*Wx^a^*) starches showed a marked shift in the endothermic peak (Figure 4A), which suggested that high AC rice tends to have a higher gelatinization temperature. The DSC parameters were then calculated and compared, as shown in Table S2. We observed that although Q11(*Wx^lv^*) and NIL(*Wx^a^*) starch showed similar DSC curves, the DSC parameters differed between these two samples. Q11(*Wx^lv^*) starch exhibited relatively higher *T*_o_, *T*_p_, and *T*_c_ but lower Δ*H* values, compared with those from NIL(*Wx^a^*) and NIL(*Wx^b^*). Studies have found that the gelatinization of starch is an endothermic process, which starts from the glass transition stage in the amorphous region, leaches from the amylose molecules, and then proceeds via dissociation of the amylopectin crystallites [38]. In addition, long amylopectin chains (e.g., DP 13–24) in starch can increase the gelatinization temperature [37,39]. Moreover, studies from non-glutinous rice found that the gelatinization properties (e.g., *T*_o_, *T*_p_, and *T*_c_) correlated positively with AC, whereas Δ*H* correlated negatively with increasing AC [18,36,40]. Thus, the enrichment of amylopectin B chains, as well as the higher AC of Q11(*Wx^lv^*) rice, led to higher *T*_o_, *T*_p_, and *T*_c_ values, but lower Δ*H* values among the three samples.

The retrogradation profiles of the gelatinized starch samples were subsequently measured using DSC. Compared with that of natural starch, all three starch samples displayed a lower retrogradation peak (Figure 4B). However, the DSC parameters differed among the three samples. Gelatinized starch from Q11(*Wx^lv^*) rice exhibited significantly higher gelatinization properties, including Δ*H*, *T*_c_, *T*_p_, and *T*_o_, than those from NIL(*Wx^a^*) and NIL(*Wx^b^*) (Table S2). Moreover, NIL(*Wx^a^*) starch also showed relatively higher Δ*H*, *T*_c_, *T*_p_, and *T*_o_ values than those of NIL(*Wx^b^*) starch. It is believed that amylose usually forms a double helix during the retrogradation process, and the outermost short branches combine to enable amylopectin crystallization [41]. Although the Q11(*Wx^lv^*) and NIL(*Wx^a^*) starches have the lowest amounts of short-branch amylopectin, they showed a relative higher Δ*H*, implying that during starch retrogradation, amylose makes a higher contribution to the formation of double-helical structures. Similar results have been found in other rice cultivars having the same genetic background [18].

## 4. Conclusions

The present study clarified the role of *Wx* allelic variation on rice grain physicochemical properties, starch structural variations, and their relationships with the physicochemical properties. Rice carrying the *Wx^lv^* allele had a higher AC but a softer GC than those carrying the *Wx^a^* and *Wx^b^* alleles. Moreover, starch fine structure analysis, determined using GPC, showed that the starch from Q11(*Wx^lv^*) rice contained significantly lower AP1 and AP2 fractions but a higher AM fraction among the three rice lines. Additionally, evidence from developing seeds suggested that the accumulation of both the AP1 and AM fractions is different among the three rice lines. Moreover, HPAEC analysis showed that Q11(*Wx^lv^*) rice contains fewer short-chain amylopectins and more intermediate chain amylopectins, resulting in decreased crystallinity and lamellar peak intensity than those of NIL(*Wx^a^*) and NIL(*Wx^b^*) rice. An assessment of the rice starch retrogradation properties, gelatinization properties, and pasting properties also revealed that the starch fine structure, as well as the starch’s ordered structure, affect these physicochemical properties. Overall, this work increased our understanding of the effects of variations of the *Wx* allele, especially *Wx^lv^*, on rice grain quality profiles between rice NILs with the same genetic background. In addition, the present study provides a resource for breeding high-amylose but low-starch-viscosity rice.

## Figures and Tables

**Table 1:** NILs-derived rice grain physicochemical characteristics.

Samples	MC (%)	PC (%)	AAC (%)	GC (mm)	TSC (%)
Q11(*Wx^lv^*)	10.76 ± 0.26a	7.14 ± 0.24a	26.58 ± 0.16a	104.75 ± 9.84a	86.44 ± 0.86a
NIL(*Wx^a^*)	11.03 ± 0.43a	7.08 ± 0.31a	25.04 ± 0.13b	36.28 ± 5.43c	86.15 ± 0.54a
NIL(*Wx^b^*)	10.92 ± 0.51a	6.98 ± 0.26a	15.42 ± 0.08c	80.56 ± 0.12b	85.92 ± 0.63a

Data represent means ± SD. In each column, a–c means values with the same letter are not significantly different (*p* ≥ 0.05). MC, moisture content; PC, protein content; AAC, apparent amylose content; GC, gel consistency; TSC, total starch content.

**Table 2:** The relative area ratios of AP1, AP2, and AM fractions of rice starch determined by GPC.

Lines	AP1	AP2	AM	AP1/AP2
Q11(*Wx^lv^*)	43.06 ± 0.09c	25.50 ± 0.03b	31.44 ± 0.06a	1.69 ± 0.00c
NIL(*Wx^a^*)	46.78 ± 0.31b	24.08 ± 0.31b	29.15 ± 0.01b	1.95 ± 0.02b
NIL(*Wx^b^*)	60.85 ± 0.14a	26.44 ± 0.12a	12.72 ± 0.27c	2.31 ± 0.00a

Data are shown as the mean ± the standard deviation. In each column, a–c means values with the same letter are not significantly different (*p* ≥ 0.05). AP1, AP2, and AM indicate the relative area ratios of each component in the total peak area of GPC, respectively. AP1/AP2 indicate amylopectin branching degree.

**Table 3:** Relative crystallinities (RC) and SAXS parameters of the NILs.

Lines	RC (%)	Imax (Counts)	D (nm)
Q11(*Wx^lv^*)	20.37 ± 0.01c	289.17 ± 3.25c	10.47 ± 0.00a
NIL(*Wx^a^*)	21.21 ± 0.06b	310.28 ± 6.68b	10.17 ± 0.04b
NIL(*Wx^b^*)	23.79 ± 0.14a	355.56 ± 10.72a	9.86 ± 0.01c

Data are shown as means ± the standard deviation. In each column, values that do not show the same letter differ significantly (*p* < 0.05). SAXS, star small-angle X-ray scattering; NILs, near-isogenic lines; Imax, lamellar peak intensity; D, lamellar distance.

## Data Availability

The data presented in this study are available on request from the corresponding author.

## Supplementary Materials

The following are available online at https://www.mdpi.com/article/10.3390/foods10123089/s1, Figure S1: Phenotypes of brown and milled rice grains from NILs, Figure S2: Crystal structure of NILs starches. Panel A shows X-ray diffraction (XRD) patterns. Panel B shows small-angle X-ray scattering (SAXS) spectra, Table S1: Pasting properties of flour and starch from NILs, Table S2: DSC determination of NILs starch thermal properties.
