# Supplementary material for: The Physicochemical Properties of Starch Are Affected by Wxlv in Indica Rice
Source: Foods. 2021 Dec 13;10(12):3089. doi: 10.3390/foods10123089 (PMC8701004; doi:10.3390/foods10123089)
Supplement: Supplementary file 1 [file foods-10-03089-s001.zip › foods-1476609-supplementary.pdf]

Supplementary Figures and Tables:

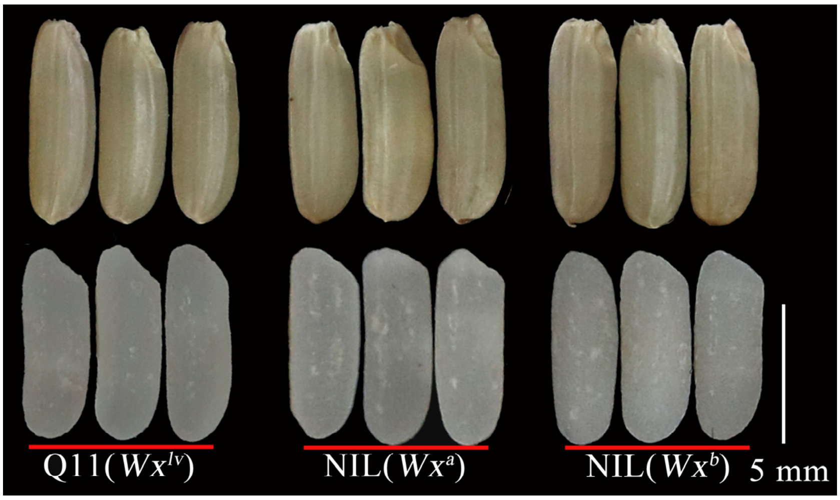

Figure S1. Phenotypes of brown and milled rice grains from different NILs.

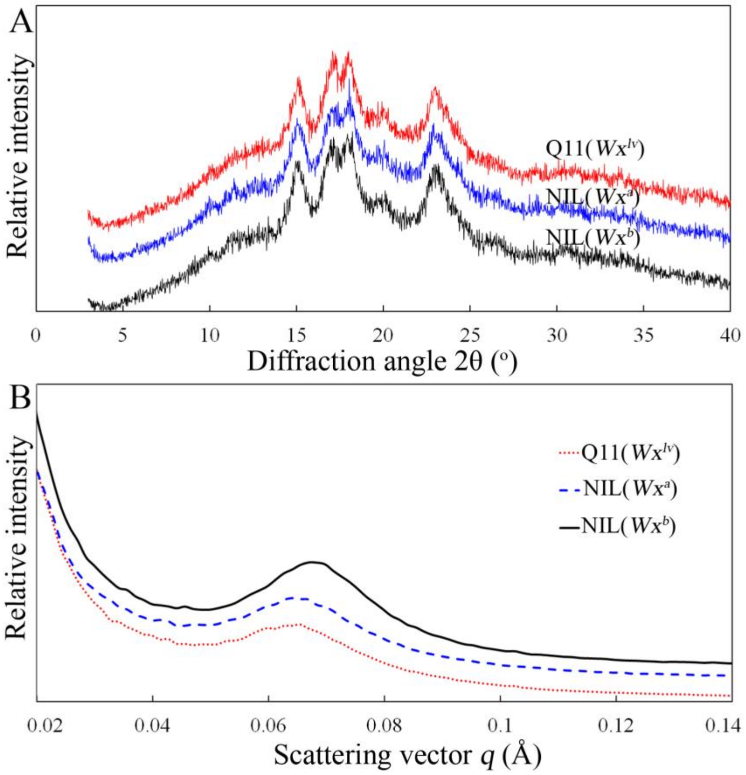

Figure S2. Crystal structure of NILs starches. Panel A shows X-ray diffraction (XRD) patterns. Panel B shows small-angle X-ray scattering (SAXS) spectra.

Supplementary Tables

Table S1. Pasting properties of flour and starch from NILs.[a]

Sample type	Lines	PKV (cP)	HPV (cP)	BDV (cP)	CPV (cP)	SBV (cP)	PT (min)
Flours	Q11(*Wx*^{lv})	839.00±13.00c	708.50±21.00c	130.50±14.00b	835.50±13.00c	-3.50±0.00b	6.33±0.00b
	NIL(*Wx*^{a})	1892.00±28.00b	1377.00±28.00b	515.00±23.00a	1592.50±33.00b	-299.50±7.00c	6.20±0.00c
	NIL(*Wx*^{b})	3022.50±35.50a	2482.50±35.00a	540.00±25.00a	3244.50±45.00a	222.00±13.00a	6.73±0.00a
Starches	Q11(*Wx*^{lv})	1725.50±38.00c	1430.00±14.00c	295.50±10.00c	2283.00±121.00c	557.50±13.00b	6.26±0.01b
	NIL(*Wx*^{a})	3114.00±71.00b	2754.50±27.00a	359.50±13.00b	3957.00±114.00a	843.00±7.00a	6.72±0.01a
	NIL(*Wx*^{b})	3761.00±22.00a	2233.00±17.00b	1528±16.00a	3188.50±49.00b	-572.50±21.00c	6.20±0.00c

[a]Data are means ± standard deviations, n = 2. Values with the same letter between the pair of *SSI* alleles in the same *Wx* background are not significantly different ($p < 0.05$). PKV, peak viscosity; HPV, hot paste viscosity; CPV, cool paste viscosity; BDV, breakdown viscosity; SBV, setback viscosity; PT, peak time.

Table S2. DSC determination of NILs starch thermal properties [a]

DSC	Lines	T_o (°C)	T_p (°C)	T_c (°C)	ΔH (J·G^{-1})
Gelatinization	Q11(*Wx*^{lv})	64.25±0.07a	70.35±0.07a	77.45±0.07a	8.79±0.11c
	NIL(*Wx*^{a})	60.55±0.07b	66.05±0.35b	72.95±0.78b	9.37±0.13b
	NIL(*Wx*^{b})	59.10±0.01c	63.40±0.00c	69.40±0.14c	11.20±0.02a
Retrogradation	Q11(*Wx*^{lv})	45.45±0.14a	54.30±0.05a	61.75±0.15a	3.49±0.13a
	NIL(*Wx*^{a})	43.05±0.01b	52.45±0.18b	60.92±0.11b	2.95±0.07b
	NIL(*Wx*^{b})	42.65±0.13c	50.50±0.42c	60.25±0.09c	2.33±0.02c

[a]Data represent means ± standard deviations. Values in the same column with different letters were significantly different (P < 0.05). T_o, onset temperature; T_p, peak temper ature; T_c, conclusion temperature; ΔH, enthalpy of gelatinization and retrogradation.
